# Supplementary material for: Biological Characterization and Genomic Analysis of Three Novel Serratia- and Enterobacter-Specific Virulent Phages
Source: Int J Mol Sci. 2024 May 29;25(11):5944. doi: 10.3390/ijms25115944 (PMC11172527; doi:10.3390/ijms25115944)
Supplement: Supplementary file 1 [file ijms-25-05944-s001.zip › Figure S1.pdf]

A

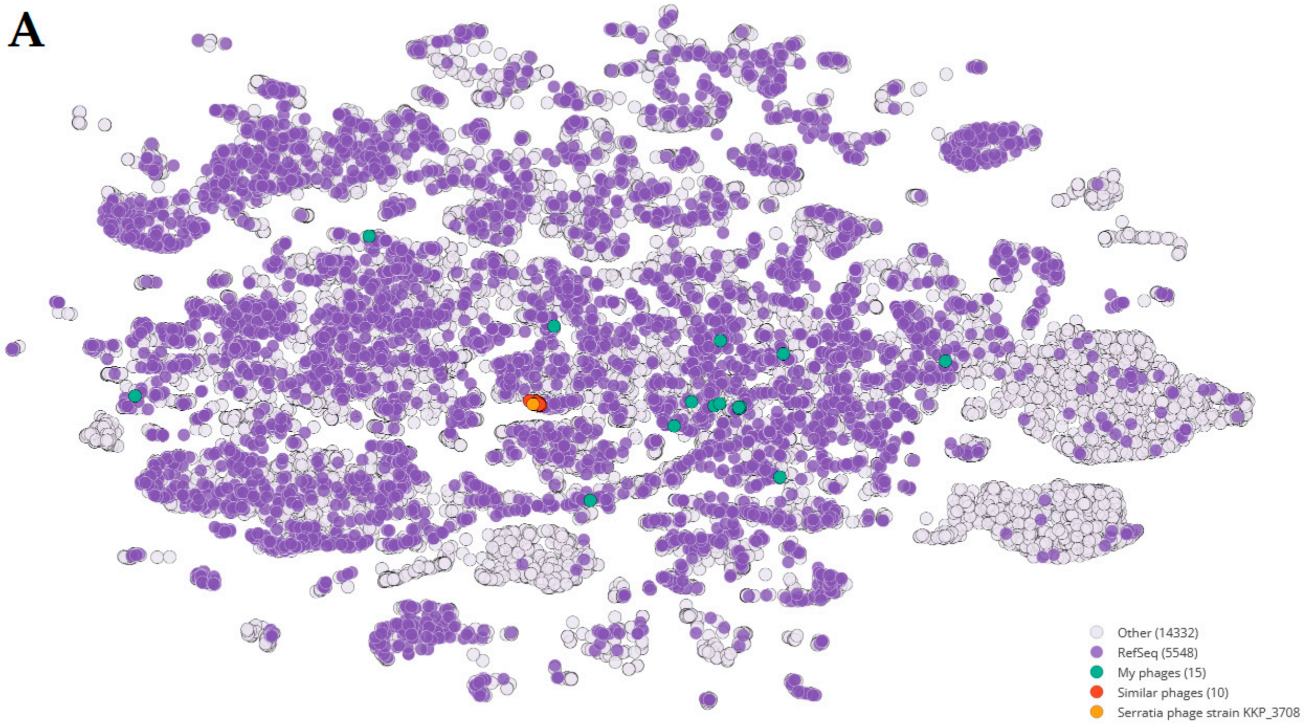
Other (14332)
RefSeq (5548)
My phages (15)
Similar phages (10)
Serratia phage strain KKP_3708

B

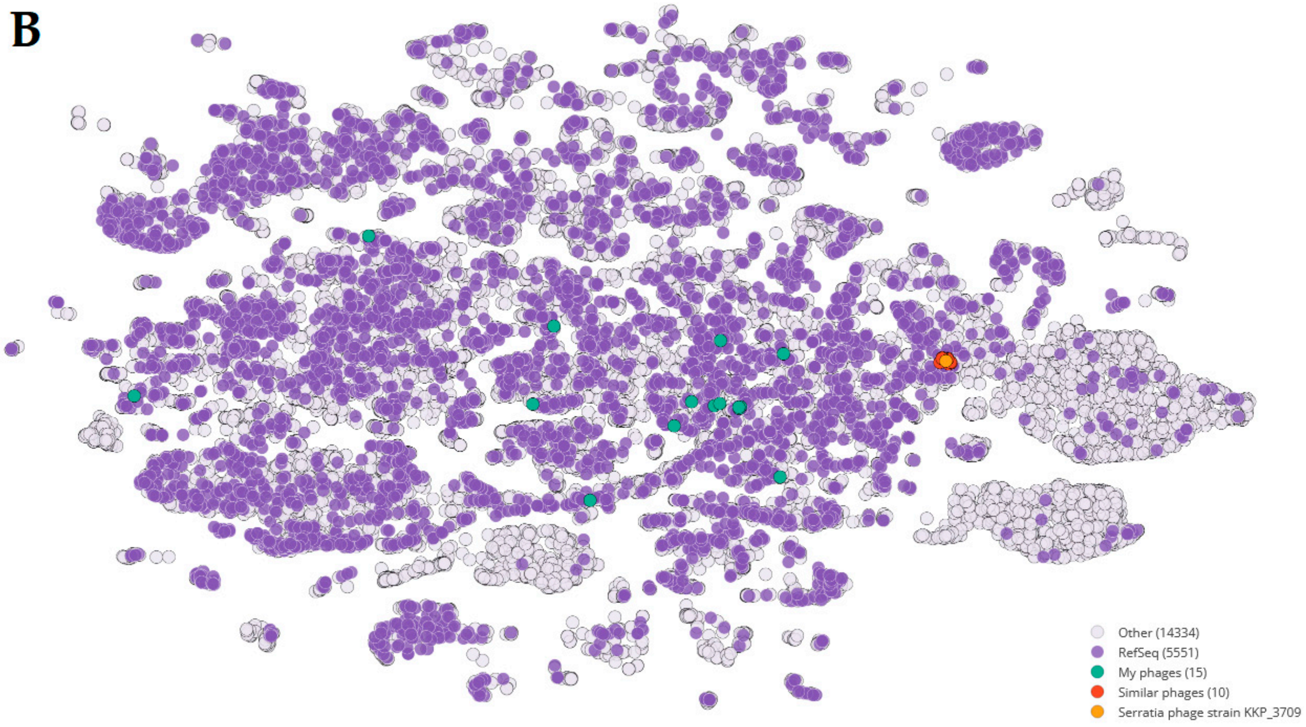
Other (14334)
RefSeq (5551)
My phages (15)
Similar phages (10)
Serratia phage strain KKP_3709

C

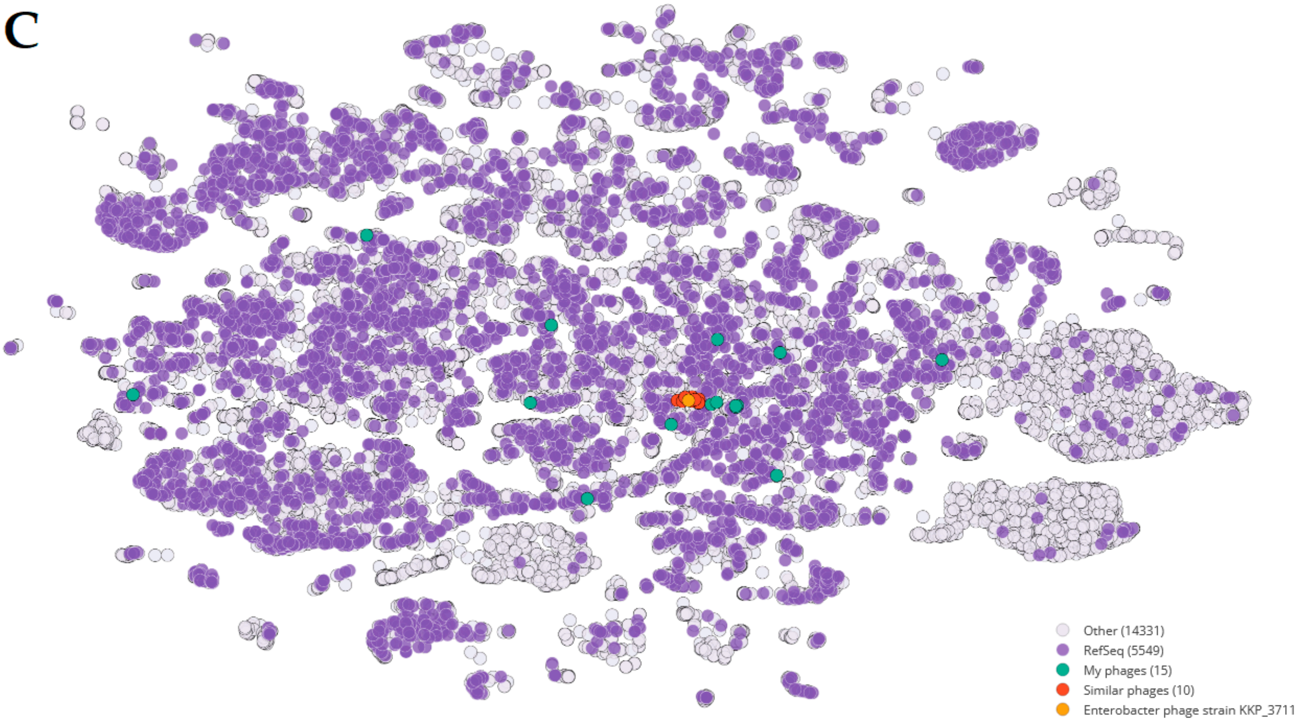

Figure S1. Phage similarity on 2D scatter plot computationally predicted and rendered through PhageAI software [89]: A – Serratia phage KKP_3708; B – Serratia phage KKP_3709; C – Enterobacter phage KKP_3711.
