# Supplementary material for: Biological Characterization and Genomic Analysis of Three Novel Serratia- and Enterobacter-Specific Virulent Phages
Source: Int J Mol Sci. 2024 May 29;25(11):5944. doi: 10.3390/ijms25115944 (PMC11172527; doi:10.3390/ijms25115944)
Supplement: Supplementary file 1 [file ijms-25-05944-s001.zip › Table S2.pdf]

Table S2. BLASTn alignment of homologs phages sequences (at the GenBank database) with isolated bacteriophages.

Phage (complete genome)	Genome Length [bp]	G+C Content [%]	No. of Genes / Protein	E–Value	Query Coverage [%]	Percent Identity [%]	Phage Family / Genus	Genome	Isolated Country	Isolation Source	Lab Host	GenBank Accession No.
**Serratia phage KKP_3708**												
Klebsiella phage Amrap	41,209	52.5	57 / 57	0.0	90	89.85	*Autographiviridae* / *Przondovirus*	Linear DNA	United Kingdom	wastewater	*Klebsiella oxytoca*	OQ579031.1
Klebsiella phage vB_Ko_K26PH128C1	41,030	52.6	51 / 51	0.0	88	92.36	*Autographiviridae* / *Przondovirus*	Circular DNA	ND	ND	ND	OY978813.1
Klebsiella phage VLCpiA3b	40,231	53.1	48 / 48	0.0	85	92.79	*Autographiviridae* / *Przondovirus*	Circular DNA	Spain	soil and water	*Klebsiella pneumoniae*	ON602742.1
Klebsiella phage P79_1	40,510	53.1	43 / 43	0.0	85	92.47	*Autographiviridae* / *Przondovirus*	Linear DNA	China	wastewater	*Klebsiella pneumoniae* strain Kp2245	OR256027.1
Klebsiella phage KP32_isolate 194	41,161	52.8	44 / 43	0.0	85	92.45	*Autographiviridae* / *Przondovirus*	Linear DNA	Russia	clinical isolate	*Klebsiella pneumoniae*	NC_047969.1
**Serratia phage KKP_3709**												
Serratia phage vB_SmaM_Hera	67,493	49.9	100 / 100	0.0	97	98.36	– / *Myosmarvirus*	Linear DNA	USA	raw sewage	*Serratia marcescens*	MW021759.1
Serratia phage MTx	68,621	49.9	103 / 103	0.0	95	97.32	– / *Myosmarvirus*	Linear DNA	USA	wastewater treatment plant sample	*Serratia marcescens*	NC_048759.1
Erwinia phage vB_EamM_TropicalSun	68,439	49.1	105 / 105	0.0	83	82.25	– / *Myosmarvirus*	Linear DNA	Indonesia	ND	*Erwinia amylovora*	MN013090.1
Serratia phage MyoSmar	68,745	49.2	105 / 105	0.0	81	83.18	– / *Myosmarvirus*	Linear DNA	USA	pond water	*Serratia marcescens*	NC_048800.1
Serratia phage SMP	68,405	49.2	99 / 99	0.0	79	82.13	– / *Myosmarvirus*	Linear DNA	China	sewage	*Serratia marcescens*	OP490597.1

**Enterobacter phage KKP_3711**												
Salmonella phage SP33	110,293	39.2	157 / 157	0.0	11	71.54	*Demerecviridae* / *Tequintavirus*	Linear DNA	China	pig farm	*Salmonella* Typhimurium	OR862218.1
Klebsiella phage vB_Kpn_3	112,003	42.1	183 / 183	0.0	9	72.05	*Demerecviridae* / *Sugarlandvirus*	Linear DNA	Iran	wastewater from Qaem hospitals	MDR *Klebsiella pneumoniae*	MZ079855.1
Escherichia phage EC142	108,723	39.0	155 / 155	0.0	9	71.75	*Demerecviridae* / *Tequintavirus*	Linear DNA	Denmark	bird feces	*Escherichia coli* strain ECOR4	ON185584.1
Escherichia phage EC104	108,711	39.0	155 / 155	0.0	9	71.75	*Demerecviridae* / *Tequintavirus*	Linear DNA	Denmark	bird feces	*Escherichia coli* strain ECOR19	ON185581.1
Escherichia phage EC122	108,723	39.0	156 / 156	0.0	9	71.75	*Demerecviridae* / *Tequintavirus*	Linear DNA	Denmark	bird feces	*Escherichia coli* strain ECOR20	ON185583.1

ND – no data
